# Coping Strategies and Health-Related Quality of Life in Individuals with Heart Failure

**DOI:** 10.3390/jcm14093073

**Published:** 2025-04-29

**Authors:** Mohammed Owayrif Alanazi, Pallav Deka, Charles W. Given, Rebecca Lehto, Gwen Wyatt

**Affiliations:** 1 Department of Nursing, College of Applied Medical Sciences, University of Bisha, P.O. Box 511, Bisha 67714, Saudi Arabia; 2 College of Nursing, Michigan State University, East Lansing, MI 48824, USA; pdeka@msu.edu (P.D.); cgiven@msu.edu (C.W.G.); lehtor@msu.edu (R.L.); gwyatt@msu.edu (G.W.)

**Keywords:** physical and emotional health-related quality of life, coping, heart failure severity

## Abstract

**Background:** Heart failure (HF) contributes to a poor physical and emotional health-related Quality of Life (HRQoL) and poor health outcomes. Coping strategies have been identified as essential in enhancing HRQoL. The study’s purpose was to examine the relationships between the factors that influence coping (i.e., age, sex, education, income, HF duration), HF severity, coping strategies (i.e., problem-focused, active emotion-focused, avoidant emotion-focused), and physical and emotional HRQoL. **Methods:** A cross-sectional study was conducted using online surveys. Descriptives, Pearson’s correlation, and one-way ANOVA analyses were used to analyze the data. **Results:** A total of 108 participants completed the study, with the majority being Black men. The result showed significant negative relationships (*p* < 0.05) between problem-focused and active emotion-focused coping and HF severity. Lower age was significantly related to the use of problem-focused and active emotion-focused coping (*p* < 0.05); females showed higher use of all coping strategies as compared with males (*p* < 0.05). A better physical HRQoL was significantly associated with active emotion-focused coping (r = −0.283, *p* = 0.005), whereas a better emotional HRQoL was significantly associated with problem-focused coping (r = −0.265, *p* = 0.005) and active emotion-focused coping (r = −0.373, *p* < 0.001). **Conclusions:** Findings showed that individuals with a lower HF severity, a younger age, and a higher income and education tended to predominantly utilize adaptive coping strategies. Individuals with HF who use problem-focused and active emotion-focused coping may experience better physical and emotional HRQoL, whereas those using primarily avoidant emotional-focused coping may need guidance in their coping strategies. Healthcare professionals may take factors such as HF severity into account to tailor interventions that promote adaptive coping and enhance HRQoL outcomes.

## 1. Introduction

Heart failure (HF) is a chronic clinical condition that negatively impacts physical and emotional health-related quality of life (HRQoL) as the disease progresses [1]. The primary goal of all physical and mental treatments for HF patients is to improve their HRQoL. In individuals with HF, HRQoL encompasses the effects of the condition on both their physical and emotional well-being [2,3,4,5]. Decrements in HRQoL are mainly attributed to the stress related to the severity of HF, e.g., symptoms (dyspnea, fatigue, palpitation, and pain), decline in physical functioning, and the overall impact of HF and the associated treatments on cognitive and emotional functioning [4,5,6,7]. The severity of HF is commonly measured by the New York Heart Association (NYHA) Classification ranging from I to IV, with IV being the most severe. Classification I is often asymptomatic, so most studies focus on categories II-IV [8]. Recent evidence suggested that coping can be an important factor in maintaining and/or improving the HRQoL of individuals with HF [9]. However, a clear understanding of the factors that affect individuals with HF coping and the relationship between the different types of coping strategies and physical and emotional HRQoL are lacking. Thus, the purpose of this study was to examine the factors that affect the stressor of HF and the impact of coping strategies on HRQoL.

This study categorized coping strategies into three main types: problem-focused, active emotion-focused, and avoidant coping [1,10,11]. First, problem-focused coping aims to directly alter the stressor. Second, active coping is defined as self-reflection strategies that attempt to manage and facilitate the emotional consequences of the stressor. Finally, avoidant coping is defined as self-distraction strategies that aim toward isolating the impact of the stressor [12,13].

When evaluating coping strategies among individuals with HF, a systematic review found that problem-focused and active emotion-focused coping strategies were associated with improvement in psychological and physical HRQoL, whereas avoidant emotion-focused coping was associated with an overall poor HRQoL and negative health outcomes [14]. Conversely, another study found that avoidant emotion-focused coping strategies could result in positive emotional HRQoL in the short-term, but long-term utilization of these strategies was more likely to result in an adverse impact on physical and emotional HRQoL [15]. These contrasting findings for the different types of coping, especially for avoidant emotion-focused coping, point to the need for further investigation.

Problem-focused and active emotion-focused coping strategies have been identified as the dominant types of coping among HF participants who were younger, male, well educated, and had a higher-income [10,16,17,18]. A longer HF duration has been associated with problem-focused coping, whereas shorter illness duration was associated with avoidant emotion-focused coping [19]. On the other hand, avoidant emotion-focused coping was predominant among HF participants who were older, female, less educated, and with lower-income [17,19].

Overall, contradictions in evidence related to coping with individuals with HF challenge the clarity regarding the contributors to HRQoL. Prominent issues include a lack of sufficient quality evidence, given that most studies are descriptive. Further, there are inconsistencies among the studies in the conceptualization and definitions of coping types, especially if emotion-focused coping was divided into active and avoidant types [2,17,20]. Also, the severity of HF stressors has mixed findings [15,21]. Such contradictions have resulted in difficulties in determining the effects of HF severity and influencing factors (such as age, sex, education, income, and HF duration) that may impact the types of coping strategies used, and how such strategies are or are not related to physical and emotional HRQoL. The present study lays the groundwork for the development of interventions to help individuals with HF build coping strategies to maintain their HRQoL. The objectives of this study were to:

1. Examine the associations between HF severity (i.e., HF classification) and the types of coping strategies (i.e., problem-focused, active emotion-focused, and avoidant emotion-focused coping).
2. Examine relationships between the influencing factors (i.e., age, sex, education, income, and HF duration) and the three types of coping strategies.
3. Identify associations between coping strategies and physical and emotional HRQoL.

## 2. Methods

This study used a cross-sectional design. After obtaining the associated University Institutional Review Board (IRB) approval, participants were recruited online using ResearchMatch.org, a secure online tool that aims to match researchers with target participants (accessed: 12 January 2023).

### 2.1. Sample and Setting

Participants who met the inclusion and exclusion criteria were identified through the ResearchMatch, an online recruitment registry supported by the US National Institutes of Health. Using convenience sampling, inclusion criteria were as follows: (1) diagnosed HF, (2) have an NYHA HF classification > I, since those with NYHA classification I are often asymptomatic; (3) age ≥ 18 years; (4) have internet access; and (5) able to speak and read English. Exclusion criteria were as follows: (1) mental or health disorders that prevented the ability to provide informed consent; (2) individuals undergoing active cancer treatment due to the potential masking of the impact of HF symptoms on the coping strategies and their effects on HRQoL. These criteria were all determined via self-report and ResearchMatch.org search filters. Using G*Power 3.1, a sample size of 74 was found to be adequate to achieve a power of 90%, with an alpha of 0.05 and an effect size of 0.30 [22]. To account for any missing or incomplete data, the target sample size was increased to 114. Effect sizes were based on previous studies evaluating coping and HF [10,20].

### 2.2. Measures

Instruments examining the study objectives captured information on NYHA classification (New York, NY, USA), influencing factors (age, sex, education, income, and HF duration), coping strategies, and HRQoL. The self-reported measures for these variables were all selected based on relevance, reliability, and validity. The following is a description of all study measures.

#### 2.2.1. HF Severity

The clinically validated NYHA classification (i.e., II, III, and IV) is a subjective measure that describes severity of the HF severity by examining symptoms, including fatigue, palpitation, dyspnea, and their associations with physical activity limitations [23,24].

#### 2.2.2. Influencing Factors

Age was measured in years and sex as male, female, or other, education was measured in five levels, where the lowest was “less than high school” and the highest was “graduate degree”, annual income was measured by five income categories, the lowest being “below $10,000–$20,000” and the highest being “$100,001 and above”. HF duration was measured in months starting from the date the patient reported that HF was first diagnosed.

#### 2.2.3. Coping Strategies

Coping strategies were measured by the BriefPl COPE (Carver, 1997; University of Miami, Miami, FL, USA) [25], a widely used measure for coping that has been used among individuals with HF [10]. Brief Cope has 28 items measuring 14 coping strategies, 2 items each coping strategy. Participants’ responses are derived on a scale of 1 to 4, where 1 refers to “I usually do not do” and 4 “I usually do.” This study classified coping strategies into problem-focused, active emotion-focused, and avoidant emotion-focused coping [25]. The reliability was 0.84 for problem-focused (3 strategies), 0.79 for active emotion-focused (5 strategies), and 0.68 for avoidant emotion-focused coping (6 strategies) [20]. The inter-scale correlation ranged from weak to high among the three coping strategies, with problem-focused and active emotion-focused showing the highest correlation (r = 0.77). However, this result was in line with the theoretical expectation that these coping strategies, which are conceptually related, would demonstrate some degree of correlation. Importantly, the conceptualization of the three coping strategies has been empirically supported [26,27].

#### 2.2.4. Health-Related Quality of Life (HRQoL)

Various tools such as the SF-36, MLHFQ, KCCQ, and MQOL are commonly used to assess the quality of life in patients with heart failure. Each of these tools evaluates different dimensions of QOL, and their applicability can vary based on the patient’s clinical profile. For example, the Minnesota Living with Heart Failure Questionnaire (MLHFQ; University of Minnesota, Minneapolis, MN, USA) is tailored specifically for HF patients, while the SF-36 offers a more general assessment across physical and mental domains. Including such tools allows for a more comprehensive understanding of treatment outcomes and helps in tailoring interventions that improve the patients’ quality of life [28,29]. The MLHFQ was used to measure HRQoL [28]. The MLHFQ items were answered on a 6-point rating scale, ranging from 0 (none) to 5 (very much), and the higher scores indicated a worse HRQoL. The MLHFQ has a physical subscale with 8 items ranging from 0 to 40 (Cronbach’s alpha of 0.86), and an emotional subscale has 5 items ranging from 0 to 25 (Cronbach’s alpha of 0.86) [28].

### 2.3. Statistical Analysis

Prior to the analysis, the missing data were assessed, and only 4 cases with missing data were identified, accounting for less than 5% of the measured variables. The pattern of missing data was missing completely at random (MCAR). The MCAR pattern was handled by using a single imputation method [30]. After checking assumptions, including the Shapiro–Wilk test for normality and Levene’s test for homogeneity of variance, Pearson’s correlations and one-way ANOVAs were conducted to examine the relationships between influencing factors (i.e., age, sex, income, education, and HF duration) and coping strategies. The associations between the coping strategies and physical and emotional HRQoL outcomes were performed using Pearson’s correlation coefficient. The Kruskal–Wallis test was used to compare the three NYHA classifications and coping strategies [31]. If the test results were significant, the Tukey’s test post hoc analysis was applied in order to identify the differences between the NYHA classification groups. Finally, Welch’s F was used when the homogeneity of variance assumption was violated due to the unequal sample sizes for each NYHA classification subgroup. Statistical analysis was performed using SPSS software (version 25) [32].

## 3. Results

### Participant Characteristics

**Figure 1:**
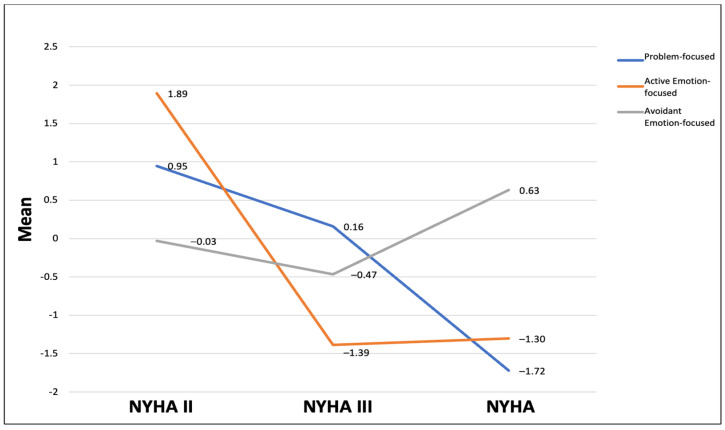
Association between coping strategies and HF severity (NYHA classification).

A total of 108 participants, who ranged in age from 20 to 81 years (37.03 ± 11.77 years), completed the study. The majority were males (57.4%, n = 62) who self-identified as Black or African American (60.2%, n = 65). Most participants were married and employed full-time (Table 1). The mean scores for problem-focused, active emotion-focused, and avoidant emotion-focused coping were presented in Table 2. However, prior to the analysis, all three types of coping strategies scores were centered on their means to reduce multicollinearity and to improve the reliability and interpretation of the test results [33]. This was achieved by subtracting the mean of the coping strategy from each participant’s score. Figure 1 illustrates the utilization of the coping strategies across the three NYHA classifications. Individuals with NYHA II used both problem-focused and active emotion-focused coping strategies. Problem-focused coping was mostly used by individuals with NYHA II (M = 0.95) and rarely used among those with NYHA IV. However, the most reported coping strategy among individuals with NYHA II was active emotion-focused coping (M = 1.89). Participants with NYHA class III showed higher use of problem-focused strategies (M = 0.16), whereas those in NYHA IV relied on avoidant emotion-focused coping, compared to those in NYHA III (M = −1.72), (Figure 1).

Using a one-way ANOVA to explore the relationship between the coping strategies and the NYHA classification, the results showed significant differences in problem-focused (F (2, 65.53) = 3.71, *p* = 0.030) and active emotion-focused (F (2, 68.48) = 3.10, *p* = 0.051) coping strategies among the NYHA classifications. To compare the specific differences between the NYHA classifications, the Games–Howell post hoc test was used. The test revealed a statistically significant negative difference in problem-focused coping between NYHA classification II and NYHA IV only (M = −2.67, *p* = 0.024), suggesting a significantly lower use of problem-focused coping among those with a higher HF severity. Active emotion-focused and avoidant emotion-focused coping did not show significant differences between NYHA II and NYHA III (M = 3.28, *p* = 0.08; M = 0.44, *p* = 0.95, respectively) or between NYHA II and NYHA IV (M = 3.19, *p* = 0.073; M = −0.66, *p* = 0.90, respectively).

The results showed that age has a significant negative correlation with problem-focused (r = −0.262, *p* = 0.006) and active emotion-focused coping (r = −0.0192, *p* = 0.046), indicating that with the increasing age, the use of these types of coping strategies tended to decrease. However, there were no significant correlations between age and avoidant emotion-focused coping (r = −0.098, *p* = 0.13). The results showed significant differences based on sex in using problem-focused (F (1, 106) = 6.72, *p* = 0.011), active emotion-focused (F (1, 106) = 5.82, *p* = 0.018), and avoidant coping (F (1, 106) = 4.77, *p* = 0.031). Those who self-identified as females tended to use more problem-focused (M = 1.21), active emotion-focused (M = 1.73), and avoidant emotion-focused (M = 1.60) coping compared to males (M = −0.90, −1.28, and −1.19, respectively). Although males had a higher mean age (M = 39.13) compared to females (M = 34.20), there were no significant differences in the relationships of age and coping according to sex. In other words, age tended to affect coping similarly for both males and females.

Annual income was significantly and positively associated with problem-focused (F (4, 103) = 2.679, *p* = 0.036), active emotion-focused (F (4, 103) = 7.067, *p* < 0.001), and avoidant emotion-focused coping (F (4, 103) = 3.290, *p* = 0.001). Analyzing the mean scores for the coping strategies within each income range showed that problem-focused coping was predominant among those who had higher annual income (i.e., $100,000 and above; M = 1.27). Furthermore, active emotion-focused coping was used mostly by individuals with $60,001–$100,000 annual income (M = 2.4). Finally, avoidant emotion-focused coping was the most frequently used coping strategy for individuals with income below $20,000 (M = 0.59). The findings suggest that individuals with HF who have lower annual income ranges tend to rely more on avoidant emotion-focused coping strategies, while those who have higher annual incomes tend to use more problem-focused and active emotion-focused coping strategies.

Level of education was significantly and positively associated with problem-focused coping (F (5, 102) = 3.4, *p* = 0.007), active emotion-focused (F (5, 102) = 8.58, *p* < 0.001), and avoidant emotion-focused coping (F (5, 16.88) = 15.51, *p* < 0.001). Post hoc analysis showed that those with the lowest level of education (i.e., completed grade school) were positively associated with all three types of coping (M = 4.16 ± 3.73, 8.17 ± 4.69, 9.13 ± 5.10, respectively). Those with a low level of education (i.e., some high school) were associated with lower scores of all coping strategies (M = −2.94 ± 4.72, 0.50 ± 5.85, −3.20 ± 3.05, respectively). Although education and income were correlated (i.e., higher income was associated with higher levels of education), there were no associations found between the higher levels of education (i.e., high school and college) and the use of problem-focused coping. Additionally, when adding income to the model, the effect of education did not change.

Finally, HF duration was not significantly related to problem-focused (r = −0.179, *p* = 0.068), active emotion-focused (r = 0.163, *p* = 0.096), and avoidant emotion-focused coping (r = −0.159, *p* = 0.106), indicating a lack of impact on coping strategies. Further, illness duration was not related to HF severity, age, and sex.

Pearson’s correlation analysis was used to explore the associations between three coping strategies and the physical and emotional domains of HRQoL measured by the MLHFQ. A significant negative correlation was found between physical HRQoL and active emotion-focused coping (r = −0.283, *p* = 0.003). However, there were no significant correlations between problem-focused (r = −0.147, *p* = 0.130) and avoidant emotion-focused coping (r = −0.108, *p* = 0.267) with physical HRQoL. Thus, only active emotion-focused coping was shown to have a significant relationship with physical HRQoL, suggesting that better physical HRQoL was associated with the use of active emotion-focused coping only. Emotional HRQoL showed significant negative correlations with both problem-focused (r = −0.265, *p* = 0.005) and active emotion-focused coping (r = −0.373, *p* < 0.001). However, there were no significant correlations between emotional HRQoL and avoidant emotion-focused coping (r = 0.019, *p* = 0.844).

The confounding effect of the NYHA classification, age, sex, education, and income among coping strategies and physical and emotional HRQoL was examined separately. Only the level of education was shown to have a small confounding effect between active emotion-focused coping and physical HRQoL. The significant negative correlation was reduced from r = −0.283 (*p* = 0.003) to r = −0.215 (*p* = 0.034). However, the correlation remained significant. No significant changes were identified on the other variables, suggesting that NYHA classification and influencing factors have no effect on the relationship between coping strategies and physical and emotional HRQoL.

## 4. Discussion

The present study examined the relationships among the stressors of HF severity and types of coping (problem-focused, active emotional-focused, avoidant emotional-focused) and the influencing factors on physical and emotional symptoms of HRQoL outcomes. The findings of this study contribute to an improved understanding of the factors that affect HF individuals’ coping and the relationships between different types of coping strategies and physical and emotional HRQoL.

The results showed significant negative associations between the severity of HF and problem-focused and active emotional-focused coping, and a positive association with avoidant emotional-focused coping strategies. The findings aligned with previous research suggest that the severity of the stressor contributes to the employed coping strategy [34]. Individuals with NYHA IV were more inclined to use avoidant emotion-focused coping as compared to those with NYHA II and III. However, those with NYHA II used problem-focused and active emotion-focused coping. Such findings suggest that individuals with a lower HF severity would have a greater ability to use more adaptive types of coping, which also has been linked to better HRQoL in the literature [1,9].

The sample came from diverse backgrounds, including relatively younger HF individuals, with ages that ranged from 20 to 81 years old with a mean age of 37.03. More than half of the participants were under 40 years of age (n = 66). Several studies have reported that older individuals with HF tend to rely more on avoidant coping strategies and less on problem-focused and active emotion-focused coping strategies [9]. However, this study obtained conflicting findings, indicating that age has a significant negative association with problem-focused and active emotion-focused coping strategies. This suggests that younger individuals with HF tend to use more problem-focused and active emotion-focused coping strategies.

The examination of the relationships between sex and coping strategies showed an interesting difference in the use of coping strategies. Most participants were male and used more problem-focused coping and less active emotion-focused coping whereas females used more active emotion-focused coping and less problem-focused coping. It is important to acknowledge that there were no significant differences between males and females in physical and emotional HRQoL. Contrary to these findings, a study from China that examined the associations between sex and coping strategies among individuals with HF (n = 360) identified that there were no sex differences in coping strategies (53.6% female) [19]. The differences found in this study may have been impacted by it being conducted within ethnic minority regions in China, which affect the generalizability of the results.

The results of this study on sex are consistent with the literature on the cardiovascular population. A study conducted on 57,017 individuals with cardiovascular disease (CVD) in Japan showed that problem-focused coping (e.g., planning) was dominantly used by males, while most women with HF used avoidant coping [18]. Similarly, a study on individuals with chronic kidney disease (n = 135) found that gender was an independent predictor of the use of avoidant coping, and this use was considerably higher in women compared to men [4]. Even though the conditions themselves differ in several aspects, such as the acute underlying pathophysiology, chronic diseases including HF, diabetes, chronic kidney disease, and COPD have overlapping features when it comes to the patient coping, particularly with regard to how individuals cope in the treatment of chronicity, emotional distress, and quality of life. Insights gleaned from comparisons between these conditions, particularly when examining specific subgroups (elderly, patients with comorbidities) may yield richer insight into coping strategies in heart failure [4,35]. Therefore, it is reasonable to consider these findings for individuals with HF.

Participants with higher educational achievements tended to have higher income [36]. Still, the results showed that a higher level of education was not found to be associated with problem-focused coping, while higher incomes were significantly associated with problem-focused and active emotion-focused coping and less use of avoidant emotion-focused coping. This is inconsistent with the prior literature that has demonstrated a relationship between problem-focused coping and higher levels of education [9,17,36]. Additionally, a lower level of education (completing high school and less) was associated with more avoidant emotion-focused coping and less problem-focused coping strategies. Low annual income tended to be associated with using more avoidant coping strategies compared to high income, where more problem and active emotion-focused coping was reported. This is also consistent with previous studies that showed avoidant emotion-focused coping to be common among HF individuals with a lower level of education. Several studies found similar findings, suggesting that income was a significant factor in selecting coping strategies [9,17,37]. Although the duration of HF was reported to be associated with the types of coping strategies used, this study found that HF duration has no association with the three types of coping [19].

Physical HRQoL in individuals with HF was significantly and negatively correlated with active emotion-focused coping only. This suggests that individuals with better physical HRQoL were more likely to use active emotion-focused coping strategies. However, problem-focused and avoidant emotion-focused coping were not significantly correlated with physical HRQoL. These findings align with a previous review that found active emotion-focused coping strategies to be linked with improvement in physical HRQoL [14]. However, previous research showed that avoidant emotion-focused coping was associated with poor HRQoL, which contradicts the present study findings that showed no relationships [14]. The discrepancy between the current findings and previous research could be attributed to differences in sample characteristics, such as age, socioeconomic status, and cultural factors, which may influence coping behaviors and perceptions of health-related quality of life. However, it is worth noting that the review reported on a combined concept of active and avoidant emotion-focused coping as ‘emotion-focused’ coping which could explain the discrepancies in findings.

Emotional HRQoL was significantly and negatively correlated with problem-focused and active emotion-focused coping strategies, indicating that better emotional HRQoL was associated with higher reported use of problem-focused and active coping strategies. This also suggests that individuals who are experiencing higher emotional distress secondary to HF (i.e., worse emotional HRQoL) tend to use fewer problem-focused and active emotion-focused coping strategies. Using a theoretically supported classification of coping strategies, this study provided further evidence for the role of coping strategies in emotional HRQoL in individuals with HF. Further, these findings are consistent with other studies that linked improved emotional HRQoL with the use of problem-focused and active emotion-focused coping strategies [9,14].

Overall, the findings of this study emphasize the importance of coping strategies in individuals with HF. The significant associations between problem-focused and active emotion-focused coping strategies and improved physical and emotional HRQoL suggest that future interventions, focused on supporting vulnerable individuals with HF who use the avoidance strategies, are needed to help them consider ways to adapt to problem-focused and active emotion-focused coping strategies to improve their HRQoL. Moreover, it is essential to assess HRQoL periodically to identify which treatment dimensions require more focus. Comprehensive care programs should address both physical rehabilitation and mental health support to enhance the overall HRQoL in HF patients.

The present study has several limitations. First, the small sample size may limit the generalizability of the findings. Second, the cross-sectional design does not allow for casual inferences between coping strategies and the quality of life over time. Additionally, the use of self-reporting tools may introduce bias, as patients’ perceptions could be influenced by external factors. Although our sample was predominantly Black males, limiting the generalizability of our results, future studies could address this by employing a stratified sampling method to capture a more diverse representation of the HF population. Given the use of online recruitment, there is a potential for selection bias, as the sample may not fully represent the broader HF population. Future research should consider using mixed recruitment strategies to minimize this bias and improve the applicability of the findings. Future research would benefit from a longitudinal design to observe changes in QOL and coping mechanisms over time and to capture more robust data on these variables.

## 5. Conclusions

This study highlights the importance of considering the severity of HF and influencing factors (i.e., age, sex, education, and income) when evaluating coping strategies. Further, the findings provide valuable insights into the associations among the three types of coping strategies and HRQoL. Understanding these relationships are essential for healthcare professionals and researchers when developing tailored interventions aimed to address the specific coping needs of individuals with HF to improve HRQoL outcomes.

## Figures and Tables

**Table 1:** Demographic characteristics of the study participants (n = 108).

Demographic Characteristics	Mean (SD)	Frequency	Percentage
Age	37.03 ± 11.77		
Sex			
Male		62	57.4
Female		46	42.6
NYHA Classification			
NYHA II		45	41.7
NYHA III		35	32.4
NYHA IV		28	25.9
Race			
Black or African American		65	60.2
White or Caucasian		42	38.9
Hispanic or Latino		1	0.9
Ethnic Background			
Hispanic or Latino		13	12.0
Non-Hispanic or Latino		95	88.0
Living Situation			
Living Alone		15	13.9
Living with Spouse		67	62.0
Living with Friend		6	5.6
Living with Relatives		20	18.5
Marital Status			
Never Married		23	21.3
Married or Living with Partner		65	60.2
Divorced or separated		9	8.3
Widowed		11	10.2
Level of Education			
Some grade school		9	8.3
Completed grade school		38	35.2
Completed high school		16	14.8
Some high school		3	2.8
Some college		16	14.8
Completed college		26	24.1
Income			
Below $10,000–$20,000		17	15.7
$20,001–$30,000		13	12.0
$30,001–$60,000		12	11.1
$60,001–$100,000		49	45.4
$100,001 and above		17	15.7
Employment Status			
Employed Full Time		60	55.6
Employed Part Time		19	17.6
Unemployed		10	9.3
Retired		15	13.9
Full time homemaker		4	3.7

**Table 2:** Descriptive statistics of coping strategies and health-related quality of life (n = 108).

Characteristics	Mean (SD)	Minimum	Maximum	Range
**Coping Strategies**				
Problem-focused	17.61 ± 4.31	11	24	6–24
Active emotion-focused	27.16 ± 6.58	14	40	10–40
Avoidant emotion-focused	25.868 ± 6.68	14	43	12–48
**HRQoL**				
Physical HRQoL	21.52 ± 6.35	8	38	0–40
Emotional HRQoL	13.32 ± 4.85	5	25	0–25
HRQoL total	34.84 ± 10.17	13	62	0–65
**HF Duration (years)**	2.95 ± 4.55	0	26	

## Data Availability

Data are contained within the article.

## Abbreviations

The following abbreviations are used in this manuscript:

HF	Heart Failure
HRQoL	Health-related Quality of Life
NYHA	the New York Heart Association
RCT	Randomized Controlled Trial
Brief COPE	Brief Coping Orientation to Problems Experienced Inventory
MLHFQ	the Minnesota Living with Heart Failure Questionnaire
